# Associations of coffee consumption with markers of liver injury in the insulin resistance atherosclerosis study

**DOI:** 10.1186/s12876-015-0321-3

**Published:** 2015-07-28

**Authors:** J. C. Dickson, A. D. Liese, C. Lorenzo, S. M. Haffner, S. M. Watkins, S. J. Hamren, J. K. Stiles, L. E. Wagenknecht, A. J. Hanley

**Affiliations:** 1 Department of Nutritional Sciences, University of Toronto, Toronto, ON Canada; 2 Department of Epidemiology and Biostatistics, University of South Carolina, Columbia, SC USA; 3 Division of Clinical Epidemiology, University of Texas Health Sciences Center at San Antonio, San Antonio, TX USA; 4 University of Texas Health Science Center, San Antonio, TX USA; 5 Lipomics Technologies, West Sacramento, CA USA; 6 Singulex, Inc., Alameda, CA USA; 7 Division of Public Health Sciences, School of Medicine, Wake Forest University, Winston-Salem, NC USA; 8 Departments of Medicine and Dalla Lana School of Public Health, University of Toronto and Leadership Sinai Centre for Diabetes, Mt Sinai Hospital, University of Toronto, 150 College Street, Room 341, Toronto, ON M5S 3E2 Canada

**Keywords:** Coffee, Caffeine, Decaffeinated, Type 2 diabetes, ALT, AST, NAFLD liver fat score, Fetuin-A, Liver enzymes, Insulin resistance atherosclerosis study

## Abstract

**Background:**

Coffee consumption has been associated with reduced risk of developing type 2 diabetes mellitus (T2DM) however, the mechanism for this association has yet to be elucidated. Non-alcoholic fatty liver disease (NAFLD) characterizes and predicts T2DM yet the relationship of coffee with this disorder remains unclear. Our aim was to investigate the associations of coffee with markers of liver injury in 1005 multi-ethnic, non-diabetic adults in the Insulin Resistance Atherosclerosis Study.

**Methods:**

Dietary intake was assessed using a validated 114-item food frequency questionnaire. Alanine aminotransferase (ALT), aspartate aminotransferase (AST) and fetuin-A were determined in fasting blood samples and the validated NAFLD liver fat score was calculated. Multivariate linear regression assessed the contribution of coffee to variation in markers of liver injury.

**Results:**

Caffeinated coffee showed significant inverse associations with ALT (*β* = −0.08, *p* = 0.0111), AST (*β* = −0.05, *p* = 0.0155) and NAFLD liver fat score (*β* = −0.05, *p* = 0.0293) but not with fetuin-A (*β* = 0.04, *p* = 0.17). When the highest alcohol consumers were excluded, these associations remained (ALT *β* = −0.11, *p* = 0.0037; AST *β* = −0.05, *p* = 0.0330; NAFLD liver fat score *β* = −0.06, *p* = 0.0298). With additional adjustment for insulin sensitivity, the relationship with ALT remained significant (ALT *β* = −0.08, *p* = 0.0400; AST *β* = −0.03, *p* = 0.20; NAFLD liver fat score *β* = −0.03, *p* = 0.27). There were no significant associations of decaffeinated coffee with liver markers.

**Conclusions:**

These analyses indicate a beneficial impact of caffeinated coffee on liver morphology and/or function, and suggest that this relationship may mediate the well-established inverse association of coffee with risk of T2DM.

**Electronic supplementary material:**

The online version of this article (doi:10.1186/s12876-015-0321-3) contains supplementary material, which is available to authorized users.

## Background

Coffee consumption has consistently been associated with a reduction in risk of developing type 2 diabetes mellitus (T2DM), however few studies have attempted to identify the pathophysiological pathway underlying this relationship [1, 2]. Exploring the impact of coffee consumption on major risk factors for T2DM will be important in uncovering potential mediating relationships underlying the observed beneficial association of coffee with this chronic disease. Non-alcoholic fatty liver disease (NAFLD) has been identified as a predictor of diabetes risk according to a meta-analysis of prospective population-based studies using transaminase concentrations [3]. In addition, three studies reported a significant association of ultrasound-identified NAFLD with incident T2DM [4–6]. However, the relationship of coffee with NAFLD remains unclear [7]. Evidence suggests a beneficial impact of coffee on the liver in individuals with alcoholic and viral liver diseases [8–11] and hepatocellular carcinoma [8, 9, 12], although the relationship of coffee with liver markers is largely unstudied in the context of liver disease of metabolic origin. Existing epidemiological studies support a beneficial impact of coffee consumption on the liver in apparently healthy populations, however the majority of these data were from studies of male populations in Asia [13–18]. A recent study of participants in the National Health and Nutrition Examination Survey (NHANES) extended these investigations to the United States and reported an inverse association of coffee consumption with liver enzymes, including alanine aminotransferase (ALT), aspartate aminotransferase (AST), gamma-glutamyl transferase (GGT) and alkaline phosphatase (ALP) [19]. Examining these relationships in a population with more extensive metabolic characterization, including insulin resistance and pre-diabetes, would be informative.

The existing literature on coffee and liver injury markers is limited in several aspects. First, little is known about the individual effects of caffeinated vs decaffeinated coffee. Xiao et al. (2014) were the first to differentiate between caffeinated and decaffeinated coffee in their examination of the independent relationships of recent intake, assessed by 24 h recall, of these coffee types with markers of liver injury [19]. Additional studies that distinguish between caffeinated and decaffeinated coffee consumption using measures that capture usual, rather than recent, intake of these coffee types are needed in order to assess the independent relationships of habitual intake of coffee with markers of liver injury. Second, previous investigations focused primarily on associations of coffee with routinely measured liver enzymes; examination of coffee’s relationship with other biomarkers of liver function and biomarker-based scores predictive of NAFLD would be instructive. Fetuin-A, a biomarker for inflammation and liver function, has been positively associated with insulin resistance and an increased risk of T2DM [20–22]. Investigations of the relationship of coffee consumption with fetuin-A have been limited to two studies which suggest a beneficial impact of coffee, but these findings need to be corroborated [20, 23]. Biomarker-based predictive scores derived from simple, non-invasive, clinical data are emerging as a practical, yet robust, method for predicting NAFLD [24, 25]. The validated NAFLD liver fat score was recently identified as the best non-invasive prediction score for identifying NAFLD when compared to similar tools [25]. To our knowledge, associations of coffee with this predictive score have yet to be examined. In light of these important knowledge gaps, the objective of the current study was to investigate the associations of usual caffeinated and decaffeinated coffee consumption with markers of NAFLD, including ALT, AST, fetuin-A, and the validated NAFLD liver fat score [24], in a multi-ethnic cohort.

## Methods

### Study population and design

The Insulin Resistance Atherosclerosis Study (IRAS) is an epidemiological investigation of the relationship of insulin resistance (IR) with cardiovascular risk factors in a large multi-ethnic population [26]. The design of IRAS has been published in detail [26]. Briefly, data collection was initiated in October 1992 with participants recruited from four geographic areas in the United States (San Antonio TX, San Luis Valley CO, Oakland and Los Angeles CA). The recruitment target was to achieve equal participation across categories of sex, ethnicity (non-Hispanic white, Hispanic, African American), age and glucose tolerance status (normal, impaired glucose tolerance, diabetes). In total, the baseline IRAS population included 1625 participants, from which written informed consent was obtained. This study was conducted in accordance with the Helsinki Declaration and the institutional review boards at Kaiser Permanente Division of Research (Oakland, CA), University of California (Los Angeles, CA), University of Texas Health Science Center at San Antonio (San Antonio, TX), the University of Colorado (Denver, CO), and the Bowman Gray School of Medicine (Winston-Salem, NC) approved the project.

For the current cross-sectional study, the population was restricted to individuals without T2DM, as defined by the 1999 World Health Organization criteria [27]. Further exclusions were made on the basis of missing liver enzyme data or extremes of energy intake, resulting in a final study population of *n* = 1005. Fetuin-A data were not available for all individuals however participant characteristics did not differ substantially between those for which fetuin-A data was available (*n* = 650) and those for which it was not (*n* = 355) (data not shown).

### Data collection

Participant assessments were conducted across two 4 h visits approximately 1 week apart and participants were asked to fast for 12 h prior to each visit [26]. Diabetes status was assessed on the first visit using a 75 g oral glucose tolerance test (OGTT). Blood was collected for fasting and 2-h glucose samples. IR was assessed at the second visit using the insulin-modified frequently sampled intravenous glucose tolerance test (FSIGTT), and insulin sensitivity (*S*_I_), was calculated using mathematical modeling methods (MINMOD version 3.0, 1994, Los Angeles, CA), as has been described in previous publications [26, 28–30]. Socio-demographic information was collected by participant report and questionnaires assessed energy expenditure, alcohol consumption and smoking behaviours [31].

### Exposure variables

Dietary intake over the past year was assessed using a validated interviewer-administered 114-item food frequency questionnaire (FFQ) modified for the IRAS from the National Cancer Institute’s Health Habits and History Questionnaire to include a number of regional and ethnic foods [26, 32]. This measure was validated in a sub-sample of the population through the administration of eight 24-h recalls administered on randomly selected days over a 1 year period. Results demonstrated desirable validity and confirmed the appropriateness of this instrument for use in this population [31]. Although the validation did not specifically address coffee, it is established that coffee drinking behaviour is accurately captured in FFQs (American women *r* = 0.8, men *r* = 0.9, respectively, vs dietary records) [33, 34].

Caffeinated and decaffeinated coffee consumption were assessed individually and participants reported on both the frequency and quantity of consumption [26]. Frequency was reported using a nine category scale with responses ranging from “never or less than 1 per month” to “six or more times per day” [35]. Individuals indicated whether their portion sizes were “small, medium or large compared with other men or women about your age” [32]. Frequency and portion size data were integrated in order to provide a single value reflective of intake by weighting the intake frequency by a factor of 0.5, 1 or 1.5 for the reported portion size of small, medium or large, respectively. Thus, one serving of an item reflects one participant-identified medium serving. Intakes of caffeinated and decaffeinated coffee were summed for a measure of total coffee. Categories of vegetable, fruit and whole grain intakes were calculated according to groupings established in previous work in IRAS [36]. Nutrient analysis of FFQ data was conducted using the HHHQ-DIETSYS analysis software (version 3.0, 1993; National Cancer Institute, Bethesda, MD, USA).

### Outcome variables

Fasting blood samples were used to determine markers of liver injury. Liver enzymes ALT and AST were measured using standard methods at the central IRAS laboratory with a Paramax PLA instrument (Baxter) [37]. Fetuin-A was determined using a sandwich-format immunoassay developed by Tethys (Tethys Bioscience, Emeryville, CA) with antibodies from R&D systems (Minneapolis, USA). The assay had a detection limit of 0.5 ng/ml and an inter-assay coefficient of variation of 11 %. NAFLD liver fat score was calculated according to the equation developed and validated by Kotronen et al. which includes fasting serum insulin, ALT, AST and presence of the metabolic syndrome (International Diabetes Federation harmonized definition) [24, 38].

### Statistical analysis

Statistical analyses were conducted using SAS version 9.2 (SAS Institute, Cary, NC). Participant characteristics were tested for differences across categories of total coffee intake. Chi-square tests were used to test differences in frequencies of categorical variables. For continuous variables, differences in means were tested using ANOVA unless the variable was highly skewed. In this case, the Kruskal-Wallis test for non-parametric variables was used.

Linear regression analyses were conducted to assess the independent relationships of caffeinated and decaffeinated coffee consumption, modeled as continuous variables, with markers of liver injury. The distributions of outcome variables were evaluated and natural log transformations were used for all outcome variables as they resulted in more normal distributions. Due to the presence of negative values, a constant of 6.4 was added to all NAFLD liver fat score data before the transformation. The independent associations of caffeinated and decaffeinated coffee consumption with outcome variables were analyzed using staged multivariate models. A base model adjusted for age, sex, and ethnicity. A second model additionally adjusted for energy intake and expenditure, BMI, smoking, alcohol consumption and education. Finally, a third model additionally adjusted for a number of dietary factors including whole grain consumption, vegetable and fruit intake, percent of energy intake from saturated and polyunsaturated fat as well as intake of the alternate type of coffee consumed (caffeinated or decaffeinated), and sugar-sweetened beverages (regular soda and lemonade/sweetened mineral water). To test for potential modifiers of the relationship between coffee consumption and markers of liver injury, interaction terms were tested for age, sex, ethnicity, BMI and S_I_, and were judged to be statistically significant if the p value was <0.05.

A sensitivity analysis was performed in which multivariate linear regression analyses were conducted with total coffee consumption as the exposure variable. Because of the well-known association between high alcohol consumption and elevations in liver transaminases, sensitivity analyses were performed in which regression models were repeated when individuals in the two highest categories of alcohol consumption were excluded (≥1 alcoholic drink/day). In addition, a fourth mechanistic regression model which additionally adjusted for insulin sensitivity was added in order to assess the contribution of S_I_ to the observed association of coffee and the outcome variables.

## Results

### Participant characteristics

Participant characteristics by categories of total coffee consumption are presented in Table 1. In this population, 79 % of individuals were consuming at least one type of coffee. Amounts of coffee consumed however were relatively modest with a median total coffee intake of 0.50 servings/day [interquartile range (IQR), 0.04-0.79]. Approximately 66 % of the population consumed caffeinated coffee while decaffeinated coffee intake was less common with only 28 % of individuals consuming this type. Ethnicity, level of education and smoking and alcohol consumption behaviours differed significantly across categories of total coffee consumption (Table 1). The highest category of total coffee consumption had the lowest proportion of participants with impaired glucose tolerance, however S_I_, BMI, waist circumference, family history of diabetes and energy expended did not differ significantly across categories of total coffee consumption. Energy intake, percent of energy from saturated fat and regular soda consumption differed significantly across categories where the highest energy intakes and saturated fat consumption were found in the highest categories of coffee consumption. Caffeinated and decaffeinated coffee consumption were inversely correlated (Spearman *r* = −0.20, *p* <0.0001). ALT and fetuin-A were inversely correlated with S_I_ (ALT Spearman *r* = −0.27, *p* <0.0001; fetuin-A Spearman *r* = −0.16, *p* <0.0001), and positively correlated with each other (Spearman *r* = 0.11, *p* = 0.0057).

**Table 1:** Participant characteristics by categories of total coffee consumption (*n* = 1005^a^)

	Total coffee consumption (servings/day)					
Variable	None	>0 to <0.5	0.5 to <1	1 to <2	≥2	*p*
n (%)	214 (21.3)	195 (19.4)	377 (37.5)	170 (16.9)	49 (4.9)	-
Age (years)	54 (47–62)	54 (47–62)	55 (48–63)	54 (48–60)	54 (46–61)	0.45
Sex, n (%)						0.07
Males	81 (37.9)	82 (42.1)	164 (43.5)	87 (51.2)	26 (53.1)	
Females	133 (62.2)	113 (57.9)	213 (56.5)	83 (48.8)	23 (46.9)	
Ethnicity, n (%)						<0.0001
Non-Hispanic white	99 (46.3)	61 (31.3)	136 (36.1)	83 (48.8)	28 (57.1)	
Hispanic	48 (22.4)	45 (23.1)	169 (44.8)	65 (38.2)	17 (34.7)	
African American	67 (31.3)	89 (45.6)	72 (19.1)	22 (12.9)	4 (8.2)	
Glucose tolerance status, n (%)						0.0215
Normal glucose tolerance	141 (65.9)	121 (62.1)	264 (70.0)	113 (66.5)	42 (85.7)	
Impaired glucose tolerance	73 (34.1)	74 (37.9)	113 (30.0)	57 (33.5)	7 (14.3)	
Highest education level completed, n (%)						0.0003
≤ Elementary	6 (2.8)	16 (8.2)	36 (9.6)	14 (8.2)	4 (8.2)	
≤ High school	61 (28.5)	48 (24.6)	151 (40.1)	45 (26.5)	16 (32.7)	
≤ College	114 (53.3)	92 (47.2)	137 (36.3)	76 (44.7)	22 (44.9)	
≤ Graduate school	33 (15.4)	39 (20.0)	53 (14.1)	35 (20.6)	7 (14.3)	
Smoking status, n (%)						<0.0001
Never	130 (60.8)	91 (46.7)	169 (44.8)	56 (32.9)	7 (14.3)	
Past	62 (29.0)	75 (38.5)	138 (36.6)	87 (51.2)	21 (42.9)	
Current	22 (10.3)	29 (14.9)	70 (18.6)	27 (15.9)	21 (42.9)	
Alcohol intake category, n (%)						<0.0001
Never drank	43 (20.1)	13 (6.7)	43 (11.4)	5 (3.0)	2 (4.1)	
Ex-drinker	47 (22.0)	23 (11.8)	46 (12.2)	24 (14.2)	6 (12.2)	
Very little	38 (17.8)	35 (18.0)	63 (16.8)	24 (14.2)	10 (20.4)	
<0.5 drinks/day	45 (21.0)	70 (35.9)	120 (31.9)	54 (32.0)	15 (30.6)	
0.5 to <1 drinks/day	15 (7.0)	23 (11.8)	39 (10.4)	20 (11.8)	4 (8.2)	
1 to <3 drinks/day	20 (9.4)	27 (13.9)	51 (13.6)	32 (18.9)	9 (18.4)	
≥3 drinks/day	6 (2.8)	4 (2.1)	14 (3.7)	10 (5.9)	3 (6.1)	
BMI (kg/m^2^)	27.3 (25.0–30.5)	27.2 (24.6–31.2)	27.5 (24.8–30.6)	27.4 (25.0–30.2)	26.5 (23.6–30.6)	0.49
Waist circumference (cm)	88.8 (80.0–97.3)	89.8 (81.8–97.0)	90.1 (81.5–98.7)	90.9 (81.7–99.1)	89.7 (79.3–97.8)	0.62
Insulin sensitivity (x 10^−4^ min^−1^[μU’ml]^−1^)	1.5 (0.8–2.8)	1.6 (0.9–2.9)	1.6 (0.9–3.0)	1.8 (0.9–2.9)	2.5 (1.1–3.8)	0.13
Family history of diabetes, n (%)	76 (35.5)	76 (39.0)	156 (41.4)	72 (42.4)	13 (26.5)	0.20
Dietary variables						
Total energy intake (kcal/day)	1736 (1321–2301)	1596 (1157–2110)	1945 (1366–2337)	1852 (1450–2471)	1902 (1334–2589)	0.0004
Total energy expended (kcal kg^−1^ year ^−1^)	13991 (12947–16047)	13815 (12869–15165)	14189 (12929–16059)	14124 (13085–15803)	14256 (12752–15441)	0.28
Whole grain consumption (servings/day)	0.7 (0.2–1.2)	0.6 (0.3–1.1)	0.6 (0.2–1.1)	0.7 (0.3–1.2)	0.5 (0.1–1.0)	0.52
% Energy from saturated fat	11.9 (9.5–14.1)	11.6 (9.0–13.9)	12.5 (10.3-14.6)	12.7 (11.0–14.8)	12.7 (10.8–14.6)	0.0003
% Energy from polyunsaturated fat	6.6 (5.4–8.1)	6.4 (5.2–7.8)	6.4 (5.3–7.7)	6.2 (5.4–7.7)	6.3 (5.3–7.6)	0.63
Regular soda consumption (servings/day)	0.03 (0.0–0.5)	0.03 (0.0–0.1)	0.03 (0.0–0.1)	0.03 (0.0–0.1)	0.07 (0.0–0.3)	0.0313
Markers of liver injury						
ALT (units/l)	17.0 (11.0–23.0)	16.0 (12.0–24.0)	16.0 (11.0–22.0)	17.0 (11.0–24.0)	15.0 (9.0–23.0)	0.40
AST (units/l)	21.0 (17.0–26.0)	21.0 (16.0–26.0)	21.0 (17.0–26.0)	21.0 (16.0-25.0)	19.0 (16.0–24.0)	0.56
Fetuin-A (μg/ml)^b^	938.9 (741.5–1256.7)	992.6 (828.4–1196.5)	974.3 (835.8–1165.7)	986.8 (839.6–1260.7)	1125.8 (903.9–1273.6)	0.52
NAFLD liver fat score^c^	−0.9 (−2.0, 0.8)	−1.1 (−2.1, 0.4)	−1.1 (−2.2, 0.3)	−1.0 (−2.2, 0.5)	−1.8 (−2.7, −0.1)	0.23

^a^Slight variation across variables

^b^*n* = 650

^c^*n* = 988

### Regression analyses

Caffeinated coffee consumption was inversely associated with ALT, AST and NAFLD liver fat score, and, in the case of ALT, there was a stronger magnitude of association across staged multivariate models (Table 2, model 3: ALT *β* = −0.08, *p* = 0.0111; AST *β* = −0.05, *p* = 0.0155; NAFLD liver fat score *β* = −0.05, *p* = 0.0293). Caffeinated coffee consumption however, showed no significant association with fetuin-A (Table 2, model 3: *β* = 0.04, *p* = 0.17). There were no significant associations of decaffeinated coffee consumption with markers of liver injury.

**Table 2:** Multiple linear regression analysis of the associations of caffeinated and decaffeinated coffee consumption with markers of liver injury

Outcome per unit increase in coffee	ALT^ab^		AST^ab^		Fetuin-A^ac^		NAFLD liver fat score^ad^	
	β (95 % CI)	*p* value	β (95 % CI)	*p* value	β (95 % CI)	*p* value	β (95 % CI)	*p* value
Caffeinated coffee								
Model 1	−0.07 (−0.13, −0.01)	0.0298	−0.05 (−0.09, −0.01)	0.0200	0.03 (−0.02, 0.09)	0.26	−0.07 (−0.12, −0.01)	0.0122
Model 2	−0.08 (−0.15, −0.02)	0.0100	−0.05 (−0.10, −0.01)	0.0148	0.05 (−0.02, 0.11)	0.14	−0.06 (−0.10, −0.01)	0.0218
Model 3	−0.08 (−0.15, −0.02)	0.0111	−0.05 (−0.10, −0.01)	0.0155	0.04 (−0.12, 0.11)	0.17	−0.05 (−0.10, −0.01)	0.0293
Decaffeinated coffee								
Model 1	−0.02 (−0.14, 0.10)	0.80	−0.03 (−0.11, 0.05)	0.42	−0.09 (−0.20, 0.02)	0.12	0.02 (−0.07, −0.12)	0.66
Model 2	−0.01 (−0.13, 0.11)	0.86	−0.03 (−0.11, 0.05)	0.44	−0.10 (−0.21, 0.02)	0.09	0.04 (−0.05, 0.13)	0.36
Model 3	−0.02 (−0.14, 0.09)	0.69	−0.04 (−0.12, 0.04)	0.36	−0.08 (−0.19, 0.04)	0.19	0.03 (−0.06, 0.12)	0.47

Model 1: Age, sex, ethnicity

Model 2: Adjusted as in model 1 + energy intake, energy expenditure, education, BMI, smoking, alcohol consumption

Model 3: Adjusted as in model 2 + whole grain consumption, vegetable intake, fruit intake, % energy from saturated fat, % energy from polyunsaturated fat, alternate coffee type, regular soft drinks, lemonade/sweetened mineral water

^a^Log transformation;^b^*n* = 1005 with slight variation across models;^c^*n* = 650 with slight variation across models;^d^*n* = 998 with slight variation across models

### Interaction and sensitivity analyses

Associations of coffee with markers of liver injury were not modified by age, sex, ethnicity, BMI or S_I_ (all *p* > 0.05).

Multivariate linear regression analyses were also conducted with total coffee as the exposure variable. For the liver enzymes, results of regression analyses with total coffee consumption were consistent with those of caffeinated coffee consumption, with significant associations of total coffee intake with both ALT and AST (Additional file 1: Table S1, model 3: ALT *β* = −0.07, *p* = 0.0177; AST *β* = −0.05, *p* = 0.0131). Total coffee consumption was not however, associated with NAFLD liver fat score (Additional file 1: Table S1, model 3: *β* = −0.04, *p* = 0.11) or fetuin-A, (Additional file 1: Table S1, model 3: *β* = 0.02, *p* = 0.47).

When the heaviest alcohol consumers were excluded, the significant inverse association of caffeinated coffee with ALT, AST and NAFLD liver fat score not only remained, but, in the case of ALT, there was a stronger magnitude of association compared to the initial analysis (Table 3, model 3: ALT *β* = −0.11, *p* = 0.0037; AST *β* = −0.05, *p* = 0.0330; NAFLD liver fat score *β* = −0.06; *p* = 0.0298). With additional adjustment for S_I_ in a fourth (mechanistic) model, the significant inverse association of caffeinated coffee with ALT remained, however the association with AST and NAFLD liver fat score was attenuated to non-significance (Table 3, model 4: ALT *β* = −0.08, *p* = 0.0400; AST *β* = −0.03, *p* = 0.20; NAFLD liver fat score *β* = −0.03, *p* = 0.27). Associations of caffeinated coffee with fetuin-A (Table 3, model 4: *β* = 0.03, *p* = 0.44), and decaffeinated coffee with all liver markers remained unchanged from the original analysis (data not shown).

**Table 3:** Multiple linear regression analysis of the association of caffeinated coffee consumption with markers of liver injury when individuals consuming ≥ 1 alcoholic drink/day were excluded

Outcome per unit increase in caffeinated coffee	ALT^ab^		AST^ab^		Fetuin-A^ac^		NAFLD liver fat score^ad^	
	β (95 % CI)	*p* value	β (95 % CI)	*p* value	β (95 % CI)	*p* value	β (95 % CI)	*p* value
Model 1	−0.10 (−0.17, −0.03)	0.0046	−0.05 (−0.10, −0.01)	0.0280	0.02 (−0.04, 0.08)	0.47	−0.07 (−0.13, −0.02)	0.0070
Model 2	−0.11 (−0.18, −0.03)	0.0037	−0.05 (−0.10, −0.00)	0.0420	0.02 (−0.04, 0.08)	0.54	−0.06 (−0.11, −0.01)	0.0189
Model 3	−0.11 (−0.18, −0.04)	0.0037	−0.05 (−0.10, −0.00)	0.0330	0.03 (−0.04, 0.09)	0.41	−0.06 (−0.11, −0.01)	0.0298
Model 4	−0.08 (−0.16, −0.00)	0.0400	−0.03 (−0.08, 0.02)	0.20	0.03 (−0.04, 0.09)	0.44	−0.03 (−0.07, 0.02)	0.27

Model 1: Age, sex, ethnicity

Model 2: Adjusted as in model 1 + energy intake, energy expenditure, education, BMI, smoking, alcohol consumption

Model 3: Adjusted as in model 2 + whole grain consumption, vegetable intake, fruit intake, % energy from saturated fat, % energy from polyunsaturated fat, decaffeinated coffee consumption, regular soft drinks, lemonade/sweetened mineral water

Model 4: Adjusted as in model 3 + insulin sensitivity

^a^Log transformation;^b^ *n* = 827 with slight variation across models;^c^*n* = 541 with slight variation across models; ^d^*n* = 821 with slight variation across models

## Discussion

NAFLD has emerged as an important predictor of T2DM [37, 39] and thus it is of interest to investigate the relationship of this disorder with coffee consumption in the context of attempting to identify a potential mediating role on the inverse relationship of coffee with T2DM. Our findings regarding ALT, AST and NAFLD liver fat score shed light on a potential mediating relationship that may underlie this inverse association [1, 2].

Results of the present cross-sectional examination of a multi-ethnic, non-diabetic cohort reveal significant inverse associations of caffeinated coffee consumption with ALT and AST, and in the case of ALT, there was a stronger magnitude of association across multivariate models and with the exclusion of individuals whose transaminase levels may be affected by alcohol consumption. Analyses also revealed an inverse relationship of caffeinated coffee with NAFLD liver fat score, a non-invasive validated tool for identifying NAFLD using routinely available clinical and laboratory data [24]. These findings are consistent with accumulating evidence demonstrating a beneficial impact of coffee consumption on the liver. Specifically, a beneficial effect of coffee on the risk of NAFLD as defined by elevated liver transaminases [40] or abdominal imaging [41, 42], a reduction in risk of fibrosis associated with coffee consumption in patients with NAFLD [43] and NASH [44], and inverse associations of coffee with ALT and GGT [11, 13–19]. Previous studies of associations of coffee with liver enzymes however have been conducted largely in Japanese male populations or among individuals with alcoholic and viral liver diseases. Investigations of associations of coffee with liver markers in liver diseases of metabolic origin (ie. NAFLD) are scarce. Our findings are consistent with the only other study of healthy individuals conducted in North America [19] and extend these investigations to a large multi-ethnic population with more complete metabolic characterization, including directly-measured insulin resistance and diabetes status assessed by OGTT. Our assessment of usual, rather than recent, intake of caffeinated and decaffeinated coffee consumption allowed us to better characterize coffee drinking behavior in this population and assess the independent relationships of habitual intake of coffee types with markers of liver injury.

Our findings suggest that caffeinated coffee’s beneficial impact on NAFLD may potentially mediate its inverse association with T2DM. The relationship of NAFLD with IR and oxidative stress is well established [45]. It is possible that the antioxidant properties of coffee may help to offset reactive oxygen species, thereby contributing to a reduction in IR and subsequent fatty acid deposition in the liver [45, 46]. Results of our fourth mechanistic model support this notion as additional adjustment for S_I_ attenuated associations of caffeinated coffee with markers of liver injury. Interestingly, for ALT, the more sensitive and liver-specific transaminase, the association with caffeinated coffee consumption remained significant in this model suggesting that this relationship is not entirely explained by S_I._

Investigations of the relationship of coffee consumption with fetuin-A are extremely limited [20, 23]. To our knowledge, only one study has examined the specific relationship of caffeinated coffee with fetuin-A which similarly reported null associations [20].

Our analyses did not identify significant associations of decaffeinated coffee with markers of liver injury, which is notable considering the inverse relationship of decaffeinated coffee with T2DM was shown to be stronger than that of caffeinated coffee in a meta-analysis [1]. It is likely that the lack of significant associations in the current analysis may be related to the generally modest consumption of decaffeinated coffee and narrow range of intakes in this population. The low level of decaffeinated coffee consumption in the current study may explain why our findings of a null association of decaffeinated coffee with fetuin-A are discordant with the only other study investigating this relationship [20]. Wedick et al. (2011) reported an inverse association of decaffeinated coffee with fetuin-A in their randomized control trial in which individuals consumed 5 6 oz cups of decaffeinated coffee/day [20]. It is possible that higher levels of consumption than reported in the current study are necessary to document this relationship. Alternatively, it is possible that null associations of decaffeinated coffee with liver markers may be explained by differing mechanistic pathways that are being impacted by these coffee types. It has been suggested for example that decaffeinated coffee may specifically benefit beta cell function rather than IR, which appears to be more strongly linked with caffeinated coffee in this cohort [35].

The current study has a number of strengths including its large sample size, multi-ethnic population and detailed measures used to assess participant characteristics including diabetes status, assessed using the OGTT, and S_I_ determined by FSIGTT. Further, a validated FFQ was used to measure usual intake of coffee and nutritional covariates. Unlike the majority of previous studies, our FFQ differentiated between caffeinated and decaffeinated coffee intake offering insight into the relationship of usual intake of each coffee type with outcome variables. The examination of multiple markers of liver injury, including liver transaminases, fetuin-A, and NAFLD liver fat score, represents a further strength of this study.

Potential limitations of the current study include the cross-sectional design as this precludes conclusions being drawn regarding the causality or the temporal nature of associations. As an observational study, there is potential for residual confounding. However extensive data were collected on potential covariates and these variables were carefully considered in the development of regression models. The reliance on participant self-report and the semi-quantitative design of the FFQ may introduce a degree of misclassification error in estimates of coffee consumption due to imprecise definitions of portion sizes. Such error, however, would likely be non-differential thus resulting in conservative estimates of associations. Additionally, NAFLD was not assessed using the gold standard liver biopsy, as this technique is not suitable for use in epidemiologic studies. Liver transaminase levels have been shown to be well correlated with directly-measured liver fat by magnetic resonance spectroscopy, and the NAFLD liver fat score is a validated, non-invasive method found to be more robust than similar predictive scores for identifying NAFLD, making these techniques appropriate alternatives to liver biopsy [24, 25, 47].

Finally, it should be emphasized that the magnitude of associations of caffeinated coffee with liver markers in the present study was modest, a finding that may be due in part to misclassification of consumption (which would dilute effect estimates, as described above), as well as the relatively modest consumption of coffee in the population, particularly in comparison to other studies investigating associations of coffee with markers of liver injury; the majority of which reported average consumption of 1 or more cups of coffee/day. In the present study, the median (IQR) of caffeinated and decaffeinated coffee intake were 0.4 (0–0.8) servings/day and 0 (0–0.03) servings/day, respectively. Conducting this study in a population in which coffee types were being consumed at higher levels may have improved our ability to detect associations of coffee with outcome variables particularly if coffee’s beneficial effects are exerted only at higher doses. This may be particularly important for decaffeinated coffee since consumption of this type was quite low in this cohort.

## Conclusions

Results of this multi-ethnic cross-sectional epidemiologic study identified significant inverse associations of caffeinated coffee consumption with liver transaminases ALT and AST, and the NAFLD liver fat score. There were no significant associations of decaffeinated coffee intake with markers of liver injury. These findings contribute to a greater understanding of the relationship of coffee consumption with liver injury and in turn, suggest that this relationship may mediate the well-established inverse association of caffeinated coffee and the risk of T2DM.

## Additional file

Additional file 1: Table S1. Multiple linear regression analysis of the association of total coffee consumption with markers of liver injury. (DOC 31 kb)

## Abbreviations

ALT: Alanine aminotransferase
AST: Aspartate aminotransferase
FFQ: Food frequency questionnaire
FSIGTT: Frequently sampled intravenous glucose tolerance test
IR: Insulin resistance
IRAS: Insulin resistance atherosclerosis study
NAFLD: Non-alcoholic fatty liver disease
OGTT: Oral glucose tolerance test
S_I_: Insulin sensitivity
T2DM: Type 2 diabetes mellitus
