# Supplementary material for: Associations of coffee consumption with markers of liver injury in the insulin resistance atherosclerosis study
Source: BMC Gastroenterol. 2015 Jul 28;15:88. doi: 10.1186/s12876-015-0321-3 (PMC4515880; doi:10.1186/s12876-015-0321-3)
Supplement: Additional file 1: Table S1. — Multiple linear regression analysis of the association of total coffee consumption with markers of liver injury. (DOC 31 kb) [file 12876_2015_321_MOESM1_ESM.doc]

**Table S1:** Multiple linear regression analysis of the association of total coffee consumption with markers of liver injury

| Outcome per unit increase in **total** **coffee** | **ALT^ab^** | | **AST^ab^** | | **Fetuin-A^ac^** | | **NAFLD liver fat score^ad^** | |
| --- | --- | --- | --- | --- | --- | --- | --- | --- |
|  | **β**  **(95% CI)** | **p**  **value** | **β**  **(95% CI)** | **p**  **value** | **β**  **(95% CI)** | **p value** | **β**  **(95% CI)** | **p value** |
| Model 1 | -0.06  (-0.12, -0.00) | 0.0345 | -0.05  (-0.09, -0.01) | 0.0114 | 0.01  (-0.05, 0.06) | 0.76 | -0.05  (-0.10, -0.00) | 0.0377 |
| Model 2 | -0.07  (-0.13, -0.01) | 0.0147 | -0.05  (-0.09, -0.01) | 0.0090 | 0.02  (-0.04, 0.07) | 0.59 | -0.04  (-0.08, 0.01) | 0.10 |
| Model 3 | -0.07  (-0.13, -0.01) | 0.0177 | -0.05  (-0.09, -0.01) | 0.0131 | 0.02  (-0.04, 0.08) | 0.47 | -0.04  (-0.08, 0.01) | 0.11 |

Model 1: Age, sex, ethnicity

Model 2: Adjusted as in model 1 + energy intake, energy expenditure, education, BMI, smoking, alcohol consumption

Model 3: Adjusted as in model 2 + whole grain consumption, vegetable intake, fruit intake, % energy from saturated fat, % energy from polyunsaturated fat, regular soft drinks, lemonade/sweetened mineral water

**^a^** Log transformation; ^b^ n= 1005 with slight variation across models; ^c^ n = 650 with slight variation across models;

^d^ n= 998 with slight variation across models
